# Cyclization of Single-Chain Fv Antibodies Markedly Suppressed Their Characteristic Aggregation Mediated by Inter-Chain VH-VL Interactions

**DOI:** 10.3390/molecules24142620

**Published:** 2019-07-18

**Authors:** Soichiro Yamauchi, Yoshihiro Kobashigawa, Natsuki Fukuda, Manaka Teramoto, Yuya Toyota, Chenjiang Liu, Yuka Ikeguchi, Takashi Sato, Yuko Sato, Hiroshi Kimura, Takeshi Masuda, Sumio Ohtsuki, Kentaro Noi, Teru Ogura, Hiroshi Morioka

**Affiliations:** 1 Department of Analytical and Biophysical Chemistry, Graduate School of Pharmaceutical Sciences, Kumamoto University, 5-1 Oe-honmachi, Chuo-ku, Kumamoto 862-0973, Japan; 2 Cell Biology Center, Institute of Innovative Research, Tokyo Institute of Technology, 4259 Nagatsuta, Midori-ku, Yokohama 226-8503, Japan; 3 Department of Pharmaceutical Microbiology, Graduate School of Pharmaceutical Sciences, Kumamoto University, 5-1 Oe-honmachi, Chuo-ku, Kumamoto 862-0973, Japan; 4 Graduate School of Engineering, Osaka University, 2-1 Yamadaoka, Suita 565-0871, Japan; 5 Institute for NanoScience Design, Osaka University, 1-3 Machikaneyama, Toyonaka 560-8531, Japan; 6 Department of Molecular Cell Biology, Institute of Molecular Embryology and Genetics, Kumamoto University, 2-2-1 Honjo, Chuo-ku, Kumamoto 860-0811, Japan; 7 CREST, JST, 4-1-8, Honcho, Kawaguchi, Saitama 332-0012, Japan

**Keywords:** single-chain Fv, aggregation propensity, sortase A, cyclic scFv, high-speed atomic force microscopy, dynamic light scattering

## Abstract

Single-chain Fv (scFv) antibodies are recombinant proteins in which the variable regions of the heavy chain (VH) and light chain (VL) are connected by a short flexible polypeptide linker. ScFvs have the advantages of easy genetic manipulation and low-cost production using *Escherichia coli* compared with monoclonal antibodies, and are thus expected to be utilized as next-generation medical antibodies. However, the practical use of scFvs has been limited due to low homogeneity caused by their aggregation propensity mediated by inter-chain VH-VL interactions. Because the interactions between the VH and VL domains of antibodies are generally weak, individual scFvs are assumed to be in equilibrium between a closed state and an open state, in which the VH and VL domains are assembled and disassembled, respectively. This dynamic feature of scFvs triggers the formation of dimer, trimer, and larger aggregates caused by the inter-chain VH-VL interactions. To overcome this problem, the N-terminus and C-terminus were herein connected by sortase A-mediated ligation to produce a cyclic scFv. Open-closed dynamics and aggregation were markedly suppressed in the cyclic scFv, as judged from dynamic light scattering and high-speed atomic force microscopy analyses. Surface plasmon resonance and differential scanning fluorometry analysis revealed that neither the affinity for antigen nor the thermal stability was disrupted by the scFv cyclization. Generality was confirmed by applying the present method to several scFv proteins. Based on these results, cyclic scFvs are expected to be widely utilized in industrial and therapeutic applications.

## 1. Introduction

**Figure 1:**
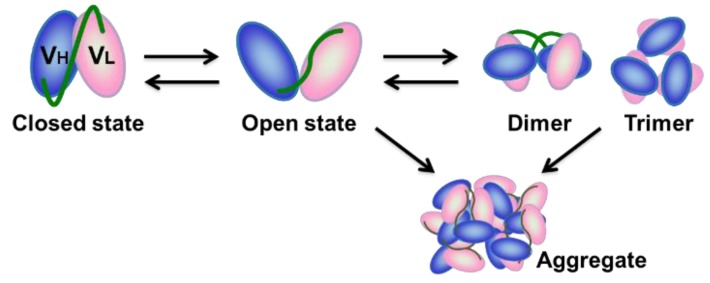
Schematic model of the open-closed dynamics and the aggregation process of single-chain Fv (scFv). ScFv protein in the open state triggers the formation of dimer, trimer, and larger aggregates mediated by inter-chain heavy chain (VH)-light chain (VL) interactions.

Monoclonal antibodies are widely used in medical and industrial applications—e.g., as therapeutic and diagnostic agents, biosensor materials, and biochemical tools [1,2,3]. In recent years, although the antibodies market has grown rapidly, monoclonal antibodies have continued to be produced using mammalian cells and advanced technologies, resulting in high production costs. Moreover, depletion of target molecules has shifted development of antibody therapeutics to that which require more sophisticated technology, for example, bispecific antibodies. Single-chain Fv (scFv) antibodies, consisting of a heavy-chain variable region (VH) and light-chain variable region (VL) connected by a short flexible polypeptide linker, are regarded as an attractive approach for overcoming these problems, since they can be produced by using *Escherichia coli* and easily engineered by genetic techniques [4,5,6,7,8]. In fact, various bispecific antibodies that use scFv as a module are developed and under clinical trials [9,10,11,12]. The use of scFvs, however, has been limited due to their inhomogeneity, which is attributable to their tendency to form dimers, trimers, tetramers, and larger oligomers [13,14,15]. Due to the weak interactions between VH and VL, the scFv protein is in equilibrium between an open state, in which the two domains are disassembled, and a closed state, in which the two domains are assembled by inter-domain interactions [13,16] (Figure 1). The open-closed dynamics of scFv is assumed be more prominent than those of IgG and Fab due to the lack of Fc regions. Accumulation of the open state scFv leads to inter-chain VH-VL interactions, resulting in the formation of oligomers [13,16]. Formation of oligomers, in turn, might increase the avidity effect, while huge oligomers or aggregates are assumed to result in precipitation. Due to lack of general methods to overcome this problem, the therapeutic scFv antibodies have not been marketed for a long time since its development around 1990 [5,17,18]. Therefore, development of a general method to suppress aggregation tendency of scFv is required for rise of these next-generation medical antibodies.

**Figure 2:**
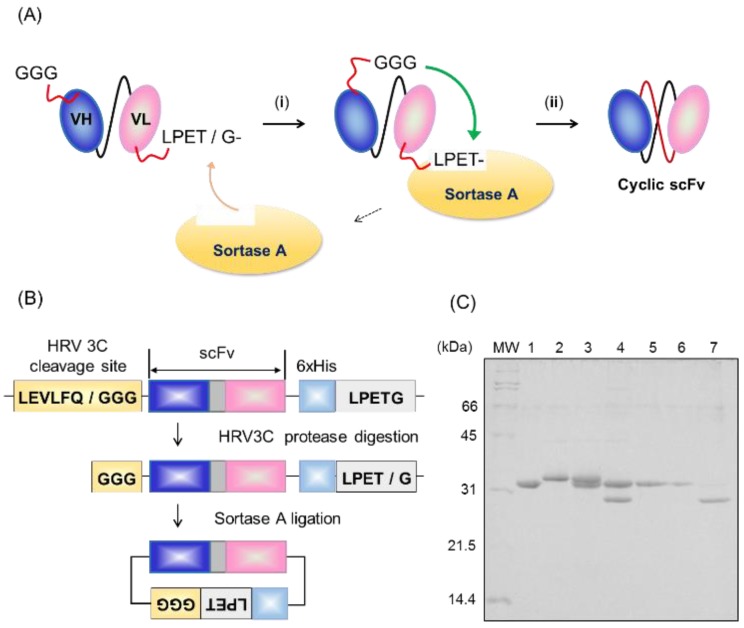
(**A**) Scheme for sortase A-mediated scFv cyclization. (i) Sortase A cleaves the peptide bond between threonine and glycine of the C-terminal LPETG motif, which leads to the formation of a covalent thioester bond between sortase A and its substrate. (ii) This thioester bond is rapidly resolved by nucleophilic attack of the N-terminal glycine residue, resulting in the generation of a cyclic scFv connected N-terminus and C-terminus covalently. (**B**) Schematic representation of the protocol for the preparation of cyclic scFv. The N-terminal tri-glycine was exposed by HRV3C protease digestion. The hexa-histidine tag was inserted in front of the LPETG motif to separate the cyclic scFv and sortase A. (**C**) SDS-PAGE analysis of sortase A-mediated scFv cyclization. Lane 1, sortase A; lane 2, 13C7-scFv-LPETG; lane 3, before sortase A reaction; lane 4, after the reaction at 25 °C for 1 h; lane 5, flow-through fraction of Ni-NTA affinity chromatography; lane 6, wash fraction; lane 7, elution fraction.

Increasing the interactions at the VH-VL interface is critical for shifting the scFv equilibrium to the closed state, in order to suppress oligomer formation. Introduction of disulfide bonds into the VH-VL interface is one of the ways to increase VH-VL interactions, in cases where the structural information is already available [19,20,21,22]. Another approach is to use the phage display system, which can be used without structural information [16,23,24,25]. These methods, however, require a large number of steps for the preparation and evaluation of a large number of mutant scFv clones. Moreover, the mutation sites and amino acid residues involved in stabilization are different among antibodies, and should be optimized for the respective scFv proteins. The development of versatile methods to suppress scFv oligomer formation would thus be helpful for the practical application of scFv proteins. In this study, to suppress the open-closed dynamics and thereby decrease the oligomer formation of scFv proteins, we designed cyclic scFvs by covalently connecting the N-terminus and C-terminus of scFvs by sortase A-mediated protein ligation techniques (Figure 2A). Sortase A, a transpeptidase found in the cell envelope of Gram-positive bacteria, cleaves the 5-residue sortase motif (LPXTG) between T and G, and connects the Gly residue exposed at the N-terminus of the protein to the C-terminus of LPXT [26,27,28,29]. We applied this method for preparation of cyclic scFv against histone H3 lysine 9 acetylation (13C7-scFv), and confirmed that the open-closed dynamics and oligomer formation were markedly suppressed in the cyclic scFv without disrupting the binding affinity for antigen and thermal stability [30]. The present method was successfully applied to several scFv proteins. The results indicate that cyclic scFv is a general and useful tool for suppressing the oligomer formation of scFv, and it can be expected to promote practical use of scFv proteins.

## 2. Results

### 2.1. Preparation of Cyclic scFv by Sortase A Ligation

For production of cyclic scFvs, we initially constructed 13C7-scFv, attached an LPETG motif at the C-terminus immediately followed by a hexahistidine tag, and an HRV3C protease cleavage site at the N-terminus to expose tri-glycine for circularization (Figure 2B and Figure S1). The 13C7-scFv was expressed in *E. coli* strain BL21 (DE3) and obtained as an inclusion body, which was solubilized by 6 M guanidine HCl (GdnHCl), purified by affinity chromatography using Ni-NTA, and refolded by gradually reducing the concentration of GdnHCl by step-wise dialysis. The refolded scFv was mixed with HRV3C protease to expose the N-terminal tri-glycine, and further purified by gel filtration column chromatography. Protein ligation was carried out at 25 °C for 1 h in 50 mM HEPES (pH 7.4), 150 mM NaCl, and 10 mM CaCl_2_. The scFv was mixed with sortase A at a molar ratio of 1:1. The intra-molecular cyclized product was observed in the SDS-PAGE band and purified by Ni-NTA affinity chromatography (Figure 2C). The prepared cyclic scFv was confirmed to be ligated at the LPETG site by mass spectrometry analysis (Figure S2).

### 2.2. Evaluation of Open-Closed Dynamics by High-Speed Atomic Force Microscopy

**Figure 3:**
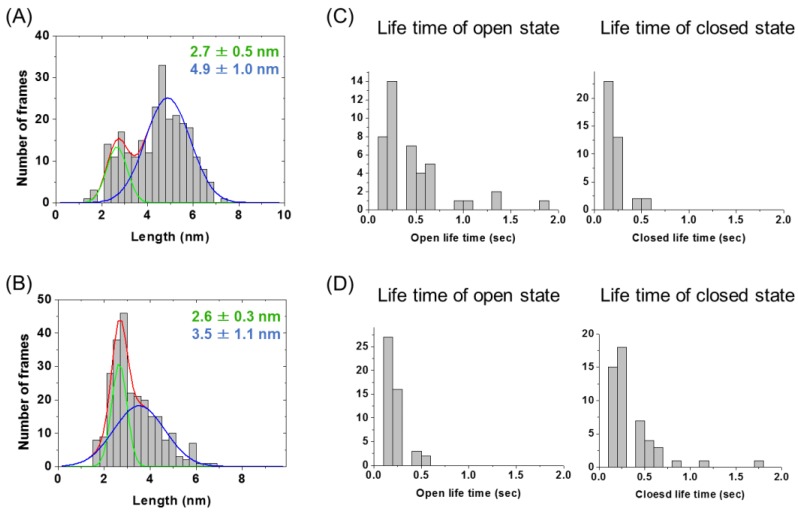
Histograms of the distance distribution between the VH and VL domains constructed from high-speed atomic force microscopy (HS-AFM) images for linear scFv (**A**) and cyclic scFv (**B**). The fitted distribution lines for all scFv molecules are shown in red, molecules with shorter distances in green, and molecules with longer distances in blue. Life time of open state (distance of over 3.5 nm between the VH and VL domain) and closed state (under 3.5 nm) for linear scFv (**C**) and cyclic scFv (**D**).

High-speed atomic force microscopy (HS-AFM) analysis was performed to evaluate the open-closed dynamics of 13C7-scFv proteins as described previously [12]. We observed the time-dependent conformational change of the linear and cyclic 13C7-scFv, and constructed histograms of the distance between the VH and VL domains from the AFM images for five scFv molecules in 5 s (Figure 3A,B and Figure S3A). In both the histogram of the linear and that of the cyclic 13C7-scFv, the molecules with longer distance (blue) and the molecules with shorter distance (green) could be observed. In the cyclic 13C7-scFv, however, the molecules with longer distance became more compact (peak at 3.5 nm) than the molecules in the linear 13C7-scFv (peak at 4.9 nm). We also observed the time-dependent changes in the distance between the VH and VL domains. We found that the dynamics of domains in the cyclic 13C7-scFv were clearly suppressed compared with that in the linear 13C7-scFv (Figure 3C,D and Figure S3B). It could be assumed that the open-closed dynamics were suppressed by cyclization of the scFv protein.

### 2.3. Measurement of Molecular Size Distributions by Dynamic Light Scattering

**Figure 4:**
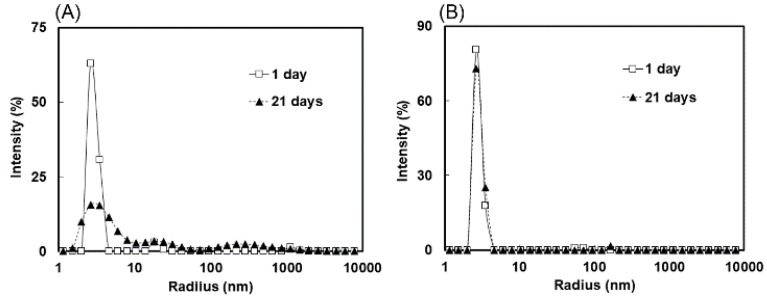
Analysis of molecular size distributions by dynamic light scattering (DLS). Linear 13C7-scFv (**A**) and cyclic 13C7-scFv (**B**) were concentrated to 5 mg/mL, and incubated at 4 °C for 1 day (solid line) or 21 days (dashed line). The DLS measurement was performed at 25 °C.

To evaluate the cohesiveness of cyclic scFv, we measured the molecular size distributions of the cyclic and linear scFvs. The cyclic and linear scFvs were concentrated to 5 mg/mL, incubated at 4 °C for 3 weeks, and then subjected to the measurement of molecular size distributions by dynamic light scattering (DLS) at 25 °C. Immediately after concentration, a single peak corresponding to the monomer was observed in both the cyclic and linear scFvs. After 3 weeks of incubation at 4 °C, the cyclic 13C7-scFv exhibited a single peak corresponding to the monomer, while appearance of the oligomer peak and reduction of the monomer peak were observed for the linear 13C7-scFv (Figure 4). DLS analysis revealed that cyclization improved the inhomogeneity of the scFv protein.

### 2.4. Evaluation of Affinity and Thermal Stability by Surface Plasmon Resonance and Differential Scanning Fluorometry

**Figure 5:**
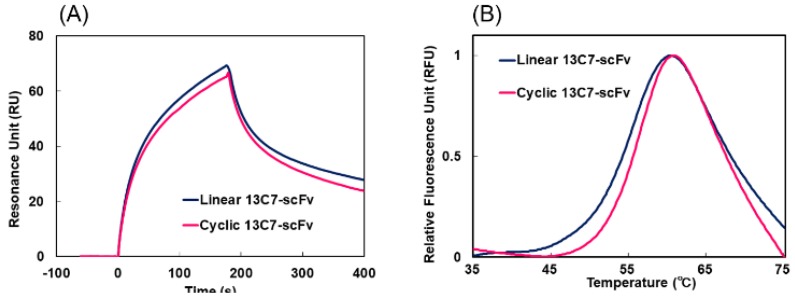
(**A**) Surface plasmon resonance (SPR) analysis of the binding affinity for the peptide derived from Lys 9 acetylated histone H3 at 25 °C for linear (blue) and cyclic 13C7-scFv (magenta). (**B**) Differential scanning fluorometry (DSF) analysis of the thermal stability of linear (blue) and cyclic 13C7-scFv (magenta).

The affinity of 13C7-scFv for antigen was evaluated by surface plasmon resonance (SPR) using a Biacore T200 instrument at 25 °C. The peptide derived from the amino acid sequences of histone H3 acetylated at Lys9, Ala-Arg-Thr-Lys-Gln-Thr-Ala-Arg-{Ac-Lys}-Ser-Thr-Gly-Gly-Lys-Ala-Pro-Arg-Lys-Gly, was immobilized on a CM5 sensor chip by amine coupling. Several concentrations of 13C7-scFv (50–400 nM) were injected at a flow rate of 50 µL/min. The shape and the intensity of the sensorgram of the cyclic and linear 13C7-scFv were similar, and exhibited highly similar *K*_d_ values of 0.32 µM and 0.19 µM for cyclic and linear scFv, respectively (Figure 5A and Figure S4). In addition, we measured the thermal stability of 13C7-scFv by differential scanning fluorometry (DSF). The melting temperature (*T*_m_) of the cyclic scFv was 57.0 °C, which was slightly higher than that of 55.6 °C for the linear scFv (Figure 5B and Table S1). These results suggest that circularization of scFv would markedly suppress aggregation without disrupting either the affinity for antigen or thermal stability.

### 2.5. Generality of Cyclization for Suppression of Aggregation of scFv Antibodies

**Figure 6:**
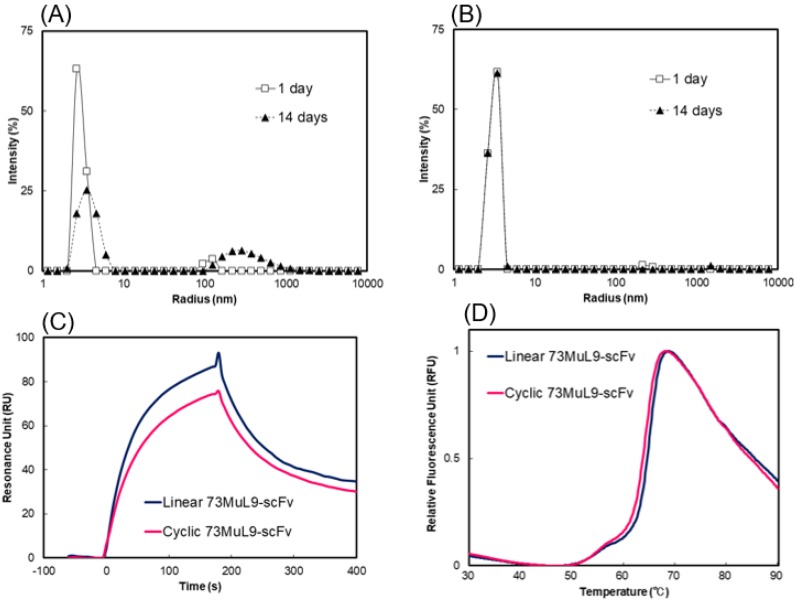
Characterization of cyclic 73MuL9-scFv. (**A**) Molecular size distributions by DLS analysis. Linear 73MuL9-scFv (**A**) and cyclic 73MuL9-scFv (**B**) were concentrated to 3 mg/mL, and incubated at 4 °C for 1 day (solid line) or 14 days (dashed line). (**C**) SPR analysis of the binding affinity for the peptide containing GA-pyridine at 25 °C for linear (blue) and cyclic 73MuL9-scFv (magenta). (**D**) DSF analysis of the thermal stability of linear (blue) and cyclic 73MuL9-scFv (magenta).

To confirm generality of cyclization for suppressing the oligomer formation of various scFv proteins, we examined this method to 73MuL9-scFv, which recognizes 3-hydroxy-4-hydroxymethyl-1-(5-amino-5-carboxypentyl) pyridinium cation (GA-pyridine), an advanced glycation end-product (AGE) that is generated by the Maillard’s reaction [16]. A method similar to that used for 13C7-scFv was applied to the preparation of cyclic 73MuL9-scFv (Figure S5). Dynamic light scattering analysis revealed that cyclic 73MuL9-scFv exhibited suppressed oligomer formation as compared to the linear 73MuL9-scFv (Figure 6A,B). Surface plasmon resonance analysis and DSF measurements revealed that neither the affinity for GA-pyridine nor the thermal stability was disturbed by cyclization of 73MuL9-scFv (Figure 6C,D, Figure S6 and Table S1). After cyclization of other scFv proteins (i.e., L1-43-scFv and TDM2-scFv [31]), reduction of oligomer formation was also confirmed by DLS, without disrupting thermal stability and binding ability for antigen (Figure S7 and Table S1). It could be assumed that the reduced oligomer formation caused by cyclization would apply to all scFv proteins.

## 3. Discussion

Here, we successfully obtained cyclic scFv by covalently connecting the N-terminus and C-terminus of scFv proteins by using a sortase A-mediated ligation technique. We showed that oligomer formation was markedly suppressed by cyclization without disturbing either the binding affinity for antigen or the thermal stability. In previous studies, cyclization was reported to increase the thermal stability and resistance to protease digestion as compared to the linear proteins, but this was not always the case in the present scFv study. Optimization of both the length and the sequence peptide linker of cyclic scFv will be required for further improvement of the thermal stability and protease resistance.

In the present study, we used the sortase A-mediated protein ligation reaction to produce a cyclized scFv antibody. Sortase A-mediated protein ligation has been widely used for both in vitro and in vivo protein engineering applications, since this reaction requires only minimal modifications of the substrate protein—namely, the addition of a C-terminal LPXTG motif and N-terminal glycine residues [32,33,34,35,36]. The attachment of this short sequence does not cause lower expression or lower solubility. Various methods, e.g., solid phase peptide synthesis, native chemical ligation, intein reaction, and enzymatic methods [37,38,39,40,41,42], have been used for the production of circular proteins. These cyclization methods could also be used for the production of cyclic scFv.

Despite the large number of antibodies that are clinically used today, depletion of target molecules has shifted development of antibody therapeutics to that which require more sophisticated technology, e.g., bispecific antibodies. Because of convenience in genetic manipulation, scFv is regarded as a promising module for development of antibody engineering. Various bispecific antibodies that use scFv as a module have been developed and are under clinical trials [9,10,11,12], and they are also undoubtedly assumed to be influenced by aggregation propensity of scFv portions. Thus, a general method to suppress aggregation tendency of scFv was assumed to contribute for development of these next-generation medical antibodies.

Plans to apply the present method to the production of scFv antibodies for medical use, e.g., scFv proteins derived from therapeutic neutralizing antibodies, antibody-drug conjugates, and bispecific antibodies, are currently underway at our laboratory. We believe that cyclization could be a versatile option to improve the inhomogeneity of fragment antibodies, and thus could promote the practical use of scFv for industrial use and medical treatment.

## 4. Materials and Methods

### 4.1. Protein Expression and Purification

The scFv genes were introduced into pCYC, modified pET-21b(+) plasmid by *Nde* I and *Not* I restriction enzyme sites (Figure S1). The hexahistidine tag at the N-terminus was followed by an HRV3C protease-cleavage site, a multiple cloning site (MCS), a hexahistidine tag, an LPETG motif, and a C-terminus hexahistidine tag. The DNA fragments coding the scFv protein were introduced into the MCS of the pCYC vector. We expressed the scFvs in *E. coli* strain BL21(DE3) cells (Novagen, Billerica, MA, USA). The transformed cells were inoculated into 300 mL of 2xYT medium containing ampicillin (100 µg/mL) and cultured at 37 °C. When the optical density at 600 nm of the cells reached 1.0, isopropyl-β-d-1-thiogalactpyranodine (IPTG) was added to a final concentration of 1 mM and the cells were cultured at 37 °C for 6 h. Then the cells were harvested by centrifugation at 4 °C and suspended in a solution of 50 mM Tris-HCl (pH 8.0) and 100 mM NaCl. The suspended cells were sonicated on ice, and the lysate was centrifuged to separate into supernatant and precipitate. The precipitate was solubilized in a solution of 6 M guanidine HCl (GdnHCl), 25 mM phosphate (pH 7.4), and 375 mM NaCl and stirred overnight at room temperature. After centrifugation (12,000 rpm, 20 min, 4 °C) to remove debris, the unfolded scFv was loaded on a Ni-NTA Superflow (WAKO, Osaka, Japan). The column was washed with a 5 column-volume of 6 M GdnHCl, 25 mM phosphate (pH 7.4), 375 mM NaCl, and 20 mM imidazole, and scFv protein was eluted with a solution of 6 M GdnHCl, 25 mM phosphate (pH 7.4), 375 mM NaCl, and 250 mM imidazole. The eluent was put in the dialysis membrane and refolded by gradually reducing the concentration of GdnHCl from 6 to 0 M. The refolded scFv was mixed with HRV3C protease, stirred overnight at 4 °C, and purified by gel filtration column chromatography using a HiLoad 16/60 Superdex 75 column (GE Healthcare, Pittsburgh, PA, USA) with a buffer solution containing 50 mM HEPES (pH 7.4) and 150 mM NaCl.

We used mutant sortase A (P94S/D160N/ D165A/K196T) for protein ligation [43]. The sortase A genes were introduced into pGBTH, a plasmid modified from pGBHPS vector [32], by *Bam* HI and *Xho* I restriction enzyme sites (Figure S8). A hexahistidine tag was inserted into the C-terminus immediately followed by a TEV protease-cleavage site. We also expressed the mutant sortase A in *E. coli* strain BL21(DE3) cells. The transformed cells were cultured using the same procedure as for scFv (induction by IPTG, followed by overnight culture at 15 °C). Then the cells were harvested by centrifugation at 4 °C and suspended in a solution of 50 mM Tris-HCl (pH 8.0) and 100 mM NaCl. The suspended cells were sonicated on ice, and the lysate was centrifuged to separate into supernatant and precipitate. The supernatant was purified by affinity chromatography using Ni-NTA. The column was washed with a 5 column-volume of 50 mM Tris-HCl (pH 8.0), 200 mM NaCl, and 20 mM imidazole, and scFv protein was eluted with a solution of 50 mM Tris-HCl (pH 8.0), 200 mM NaCl, and 250 mM imidazole. The eluate was mixed with TEV protease and stirred overnight at 4 °C to remove the hexahistidine tag, and purified by gel filtration column chromatography using a HiLoad 16/60 Superdex 75 column with a buffer solution containing 50 mM HEPES (pH 7.4) and 150 mM NaCl.

### 4.2. Protein Ligation and Purification of the Product

Protein ligation was carried out in 50 mM HEPES (pH 7.4), 150 mM NaCl, and 10 mM CaCl_2_, and then the scFv was mixed with sortase A at a molar ratio of 1:1 and incubated at 25 °C for 1 h. The resultant samples were purified by affinity chromatography using Ni-NTA. The Ni-NTA column was washed with a solution of 50 mM Tris-HCl (pH 8.0), 500 mM NaCl, and 20 mM imidazole, and the scFv protein was eluted with a solution of 50 mM Tris-HCl (pH 8.0), 500 mM NaCl, and 250 mM imidazole. The eluted sample was further purified by gel filtration column chromatography using a HiLoad 10/300 Superdex 200 column (GE Healthcare) with a buffer solution containing 50 mM HEPES (pH 7.4), 150 mM NaCl.

### 4.3. Mass Spectrometry (LC-MS/MS)

Proteins were dissolved in 100 mM Tris-HCl (pH 9.0) containing 12 mM sodium deoxycholate (SDC) and 12 mM sodium lauroyl sarcosinate (SLS). The sample solutions were reduced with 10 mM dithiothreitol (DTT) at room temperature for 30 min, and alkylated with 50 mM iodoacetamide (IAA) in the dark at room temperature for 30 min. The samples were five-fold diluted with 50 mM ammonium bicarbonate and digested with Glu-C overnight. SDC and SLS were removed by the phase transfer method [44,45]. Peptides were purified with SDB-XC StageTip [46,47]. SDC, SLS, DTT, IAA, trifluoroacetic acid, and ethyl acetate were purchased from Fujifilm Wako Pure Chemical Corporation (Osaka, Japan). Glu-C was from Promega Corp. (Madison, WI, USA).

A TripleTOF 5600 (Sciex, Framingham, MA, USA) and Ultimate 3000 RSLC nano (Thermo Fisher Scientific, Waltham, MA, USA) were employed for nanoscale liquid chromatography coupled to tandem mass spectrometry (nanoLC-MS/MS) measurement. The injection volume was 5 μL, and the flow rate was 300 nL/min. LC was performed with Acclaim PepMapTM RSLC (75 μm × 25 cm, C18, 2 μm; Thermo Fisher Scientific). The mobile phase consisted of (A) 0.1% formic acid and (B) 0.1% formic acid in acetonitrile. A linear gradient of 2% to 30% B in 22 min, 30% to 80% B in 2 min, and 80% B for 5 min was performed. A spray voltage of 2300 V was applied. The MS scan range was *m/z* 300–1250 for a precursor scan with 250 ms. The top 20 precursor ions with charge stats from +2 to +5 were selected, and the product scan of 50 ms was performed within the range of *m/z* 100–1600.

For protein identification and quantification, the MS/MS data were analyzed by ProteinPilot Software version 4.5 (Sciex, Framingham, MA, USA) with the Paragon algorithm with the database containing linear and cyclic 13C7-scFv sequences as a reference. The false discovery rate (FDR) was estimated by searching against a decoy database generated using a randomization reference database. For identification and quantification, peptides were filtered at FDR less than 1%.

### 4.4. Dynamic Light Scattering (DLS)

The DLS measurements were conducted using DynaPro NanoStar system (Wyatt Technology, Santa Barbara, CA, USA) at a wavelength of 662 nm. The distributions of molecular size were analyzed by DYNAMICS V7 software (Wyatt Technology, Santa Barbara, CA, USA). Samples were concentrated to 3 mg/mL or 5 mg/mL, and incubated at 4 °C. The samples were centrifuged (15,000 rpm, 30 min, 4 °C) immediately before measurement and kept on ice until the time of measurement. For each sample, we measured 4 µL of solution into a disposable cuvette at 25 °C, and set the acquisition time to 5 s and the number of acquisitions to 10 times for each measurement.

### 4.5. High-Speed Atomic Force Microscopy (HS-AFM)

AFM observations were performed as described previously [16].

### 4.6. Surface Plasmon Resonance (SPR)

All the SPR measurements were performed at 25 °C by using a Biacore T200 system (GE Healthcare). For SPR analysis of 13C7-scFv, the peptide derived from the amino acid sequences of histone H3 acetylated at Lys 9, A-R-T-K-Q-T-A-R-{Ac-K}-S-T-G-G-K-A-P-R-K-G, was immobilized on a CM5 sensor chip (GE Healthcare) by amine coupling with a running buffer of HBS-EP (10 mM HEPES pH 7.4, 150 mM NaCl, 3 mM EDTA, and 0.005% Tween 20) at a flow rate of 5 µL/min. A series of various concentrations of the scFv solutions were injected into the antigen-immobilized sensor chip under a continuous flow rate of 50 µL/min. The sensorgrams were normalized by subtracting the response from a reference cell, on which the peptide without acetylation, A-R-T-K-Q-T-A-R-{K}-S-T-G-G-K-A-P-R-K-G, was immobilized. After each measurement, the sensor chip was regenerated by two injections of 100 mM HCl for 18 s each, and equilibrated with running buffer for 180 s. By using Biacore T200 evaluation software version 3.0 (GE Healthcare), kinetic rate constants were calculated by globally fitting the collected data to the two-state binding model.

Surface plasmon resonance analysis of L1-43-scFvs was performed similarly as for the 13C7-scFv proteins, with slight modification. The OVA-conjugated analogue of PCB126 (Figure S8) was immobilized on a CM5 sensor chip (GE Healthcare) by amine coupling with a running buffer of HBS-EP, 1% DMSO. The sensor chip was regenerated by two injections of NaOH Buffer (10 mM NaOH, 10% DMSO, 0.5% Tween20) followed by an additional single injection of HCl Buffer (10 mM HCl, 0.5% Tween20). Kinetic rate constants were calculated by fitting to a single-site binding model.

Surface plasmon resonance analysis of 73MuL9-scFv and TDM2-scFv was performed using a Biacore T200 system (GE Healthcare, Pittsburgh, PA, USA) as described previously [16,31].

### 4.7. Differential Scanning Fluorometry (DSF)

For DSF measurement, we measured from 25–95 °C at a heating rate of 1.0 °C/min using a CFX Connect Real-Time PCR System (Bio-Rad, Hercules, CA, USA) as described previously [48,49]. All measurements were conducted using an scFv concentration of 5 µM mixed in a solution of 50 mM HEPES (pH 7.4), 150 mM NaCl and SYPRO Orange fluorescent dye (diluted 1:1000; Sigma Aldrich).

## Supplementary Materials

The following are available online at https://www.mdpi.com/1420-3049/24/14/2620/s1, Figure S1: Plasmid map of pCYC; Figure S2: Identification of the 13C7-scFv junction peptides; Figure S3: HS-AFM images of linear 13C7-scFv and cyclic 13C7-scFv; Figure S4: SPR sensorgrams of linear 13C7-scFv and cyclic 13C7-scFv at several scFv protein concentrations, and their *K*_D_ values; Figure S5: SDS-PAGE analysis of sortase A-mediated scFv cyclization; Figure S6: SPR sensorgrams of linear 73MuL9-scFv and cyclic 73MuL9-scFv at several scFv protein concentrations, and their *K*_D_ values; Figure S7: SDS-PAGE analyses of sortase A-mediated L1-43-scFv and TDM2-scFv cyclizations, and functional characterization of cyclic L1-43-scFv and cyclic TDM2-scFv; Figure S8: Plasmid map of pGBTH; and Table S1: *T*_m_ values of various scFv proteins estimated from DSF data.
