# Supplementary material for: Cyclization of Single-Chain Fv Antibodies Markedly Suppressed Their Characteristic Aggregation Mediated by Inter-Chain VH-VL Interactions
Source: Molecules. 2019 Jul 18;24(14):2620. doi: 10.3390/molecules24142620 (PMC6681014; doi:10.3390/molecules24142620)
Supplement: Supplementary file 1 [file molecules-24-02620-s001.pdf]

Supplementary Materials

Cyclization of single-chain Fv antibodies markedly suppressed their characteristic aggregation mediated by inter-chain VH-VL interactions

Soichiro Yamauchi, Yoshihiro Kobashigawa, Natsuki Fukuda, Manaka Teramoto, Yuya Toyota, Chenjiang Liu, Yuka Ikeguchi, Takashi Sato, Yuko Sato, Hiroshi Kimura, Takeshi Masuda, Sumio Ohtsuki, Kentaro Noi, Teru Ogura and Hiroshi Morioka

Table S1. T_m values of various scFv proteins estimated from DSF data

	13C7-scFv		73MuL9-scFv		L1-43-scFv		TDM2-scFv	
	Linear	Cyclic	Linear	Cyclic	Linear	Cyclic	Linear	Cyclic
T_m (°C)	55.6 ± 0.3	57.0 ± 0.6	65.1 ± 0.1	64.3 ± 0.1	52.6 ± 0.0	52.0 ± 0.0	48.2 ± 0.0	48.2 ± 0.0

Supporting Figures

Figure S1

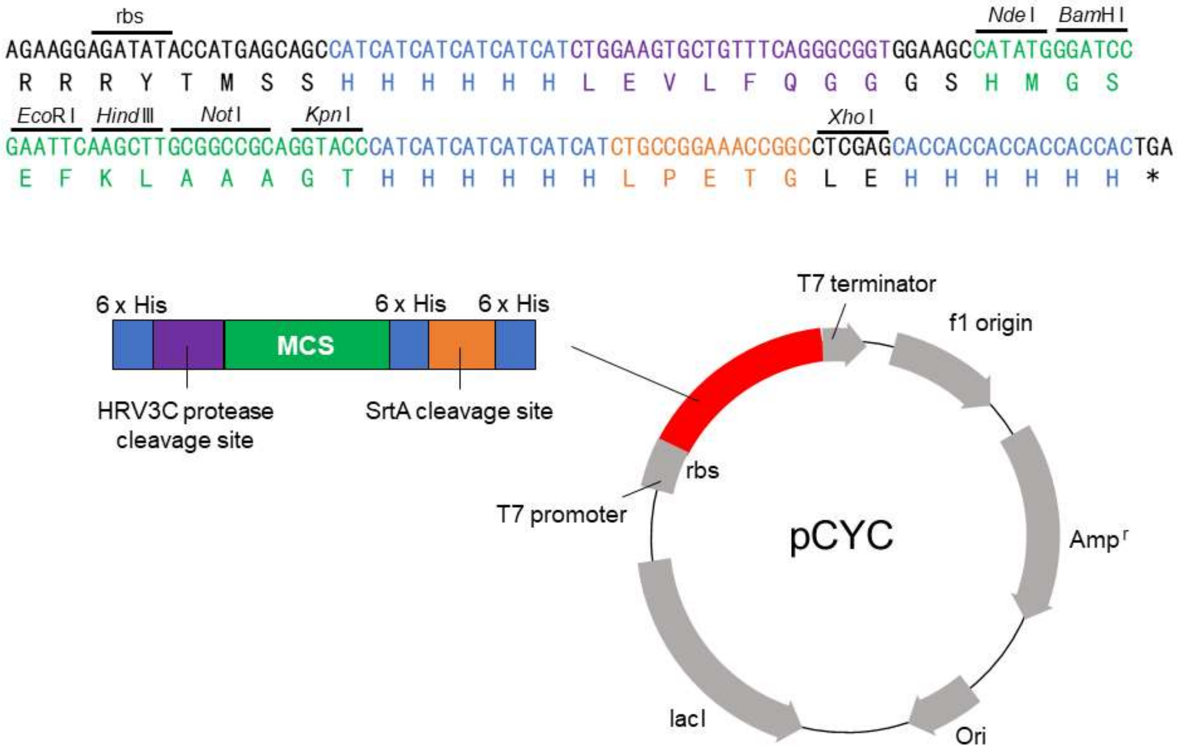

Figure S1. Plasmid map of pCYC.

Figure S2

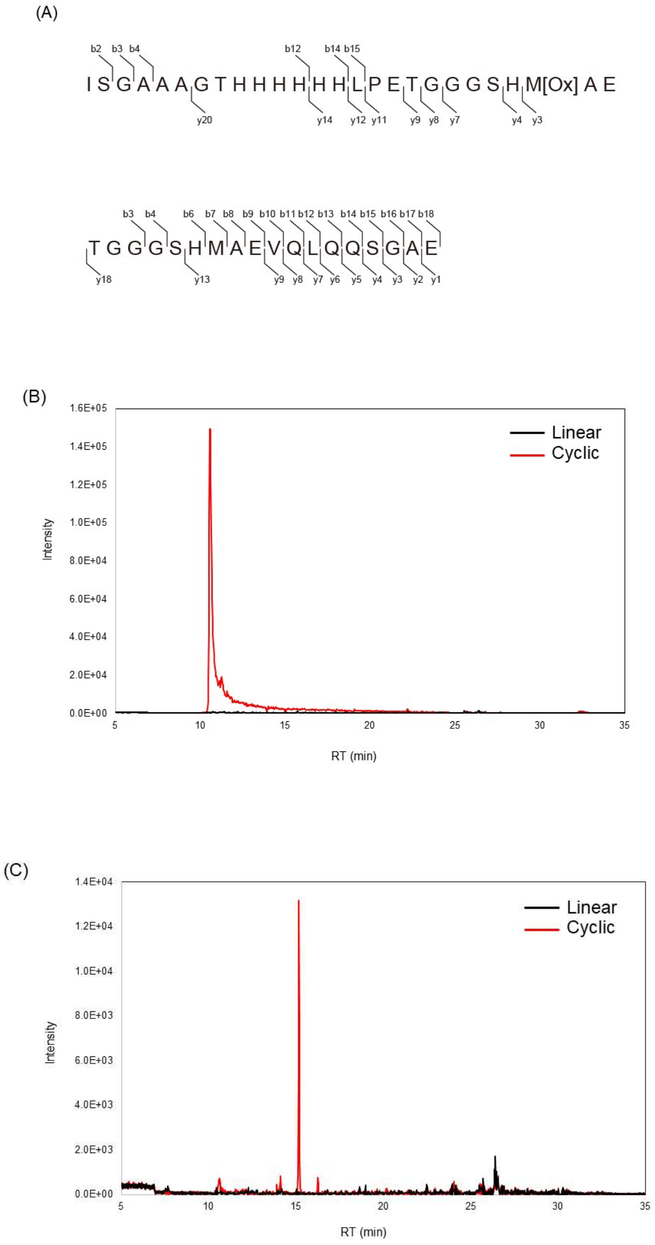

Figure S2. Identification of the 13C7-scFv junction peptides.

The 13C7-scFv junction peptides ISGAAAGTHHHHHHLPETGGGSHMAE (*m*/*z* 663.8065, z = 4) and TGGGSHMAEVQLQQSGAE (*m*/*z* 893.9063, z =2) were identified only in the cyclized sample. Observed b- or y-ions from each peptide are shown in (A). [Ox] indicates oxidation. The extracted ion current chromatograms of ISGAAAGTHHHHHHLPETGGGSHMAE and TGGGSHMAEVQLQQSGAE are shown in (B) and (C), respectively.

Figure S3

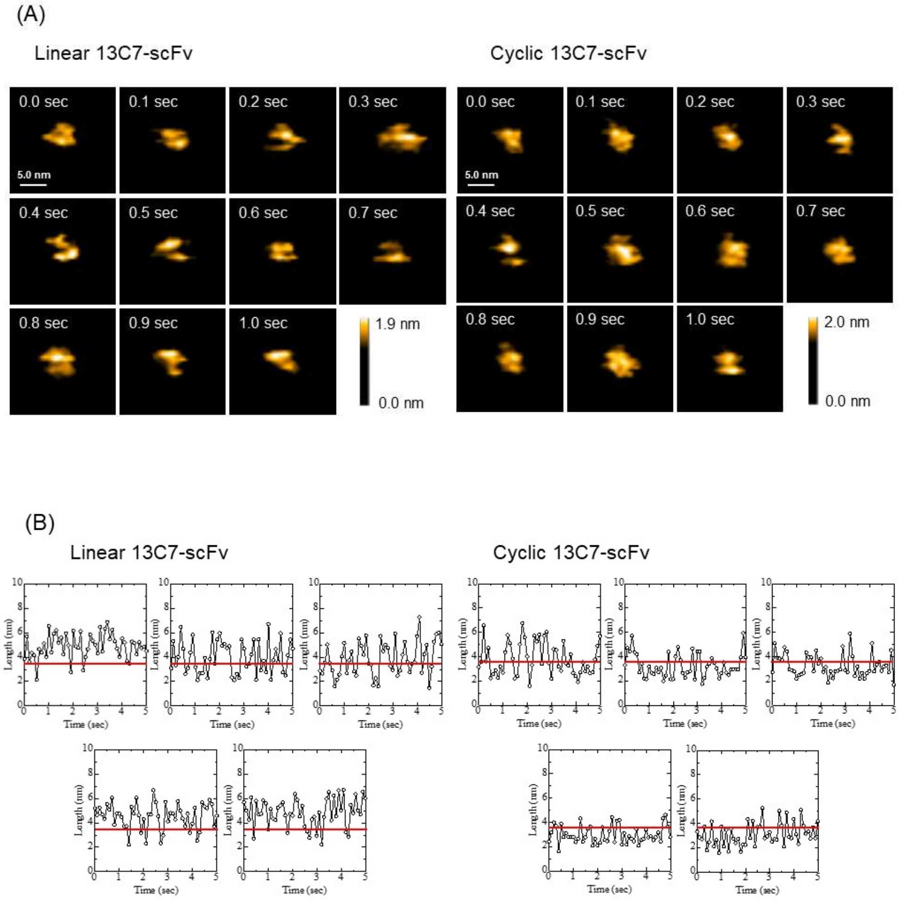

Figure S3. HS-AFM images of linear 13C7-scFv and cyclic 13C7-scFv.

(A) AFM images (20 x 20 nm) of linear 13C7-scFv (left) and cyclic 13C7-scFv (right). (B) Time-dependent change in the distance between the VH and VL domain of linear 13C7-scFv (left) and cyclic 13C7-scFv (right).

Figure S4

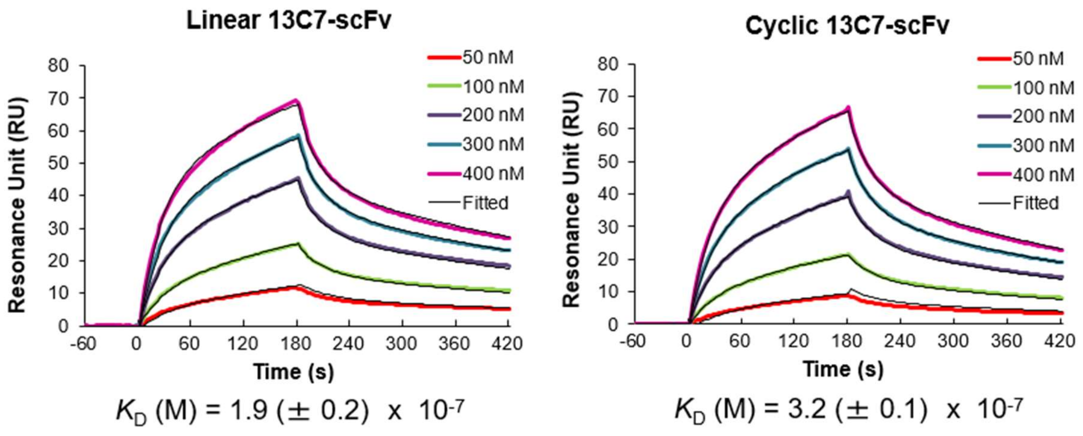

Figure S4. SPR sensorgrams of linear 13C7-scFv (left) and cyclic 13C7-scFv (right) at several scFv protein concentrations, and their K_D values.

Figure S5

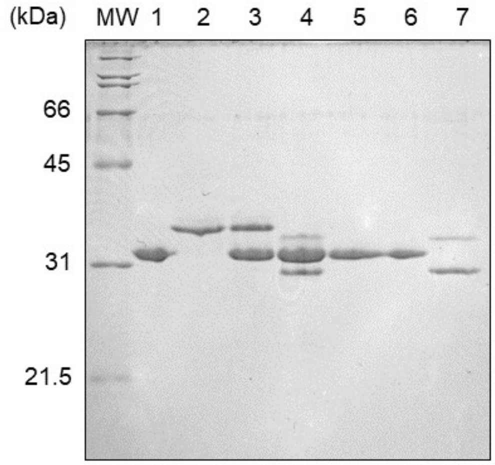

Figure S5. SDS-PAGE analysis of sortase A-mediated scFv cyclization.

Lane 1, sortase A; lane 2, 73MuL9-scFv-LPETG; lane 3, before sortase A reaction; lane 4, after the reaction at 25°C for 1 hour; lane 5, flow-through fraction of Ni-NTA affinity chromatography; lane 6, wash fraction; lane 7, elution fraction.

Figure S6

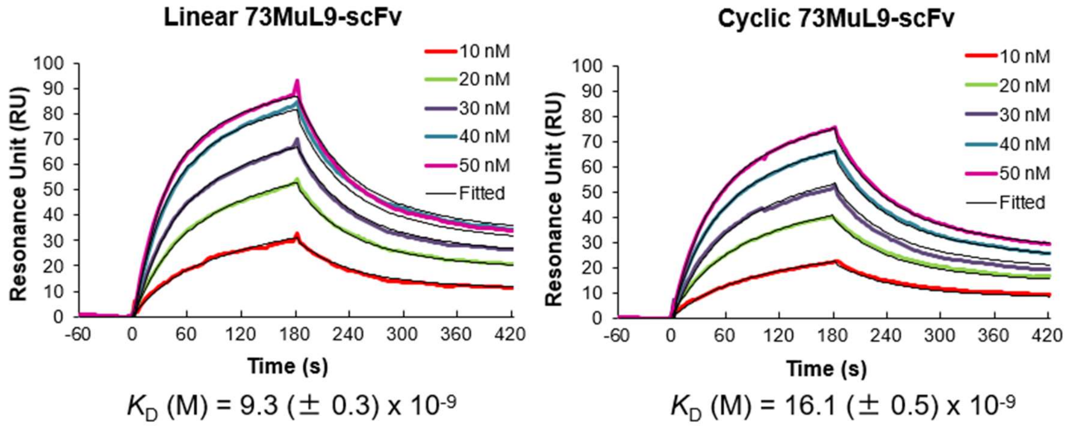

Figure S6. SPR sensorgrams of linear 73MuL9-scFv (left) and cyclic 73MuL9-scFv (right) at several scFv protein concentrations, and their K_D values.

Figure S7

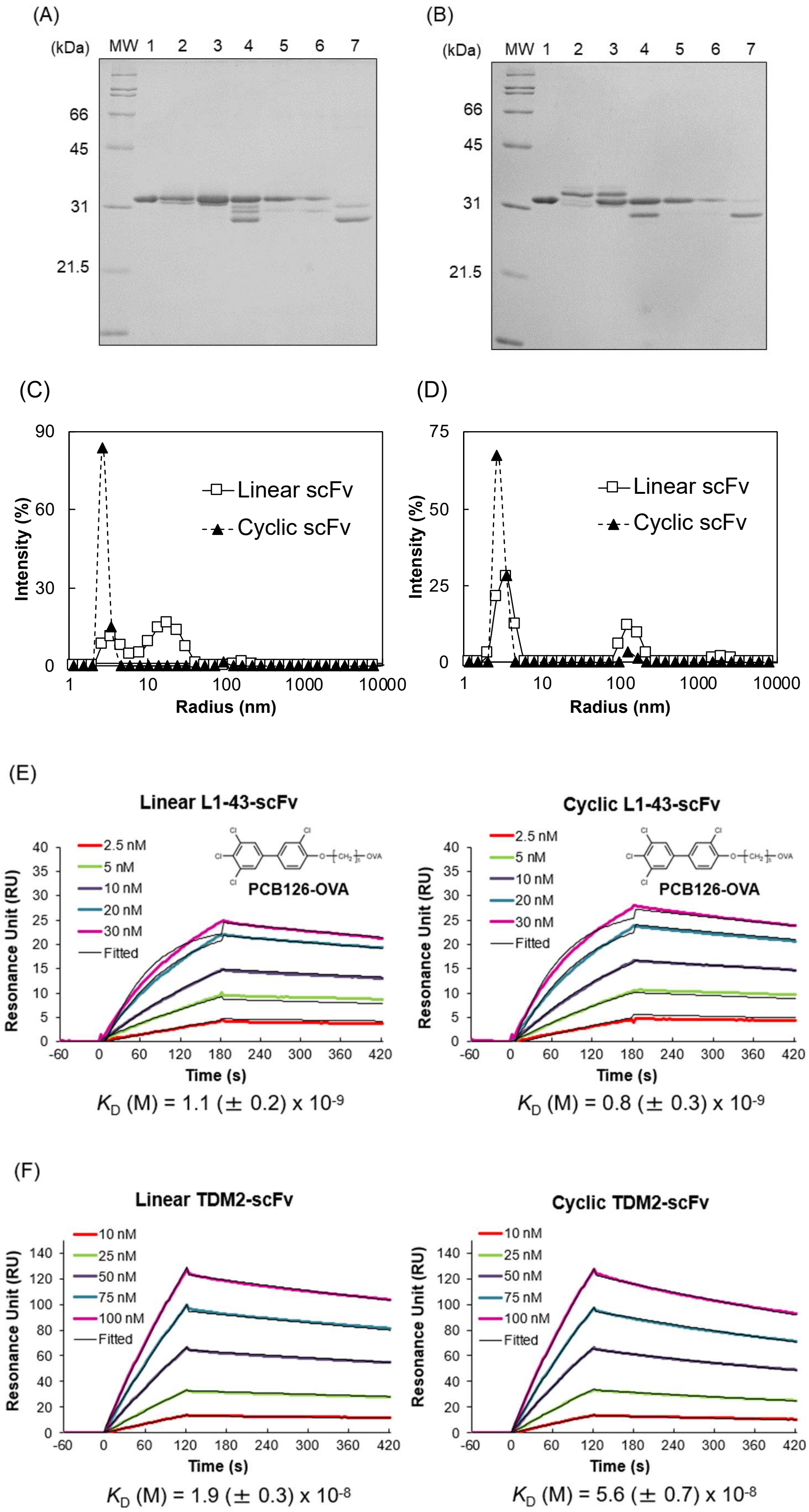

(A)
(kDa)
MW 1 2 3 4 5 6 7
66
45
31
21.5
(B)
(C)
Intensity (%)
Radius (nm)
Linear scFv
Cyclic scFv
(D)
(E)
Linear L1-43-scFv
Cyclic L1-43-scFv
2.5 nM
5 nM
10 nM
20 nM
30 nM
Fitted
PCB126-OVA
Resonance Unit (RU)
Time (s)
K_D (M) = 1.1 (± 0.2) x 10^{-9}
K_D (M) = 0.8 (± 0.3) x 10^{-9}
(F)
Linear TDM2-scFv
Cyclic TDM2-scFv
10 nM
25 nM
50 nM
75 nM
100 nM
Fitted
K_D (M) = 1.9 (± 0.3) x 10^{-8}
K_D (M) = 5.6 (± 0.7) x 10^{-8}

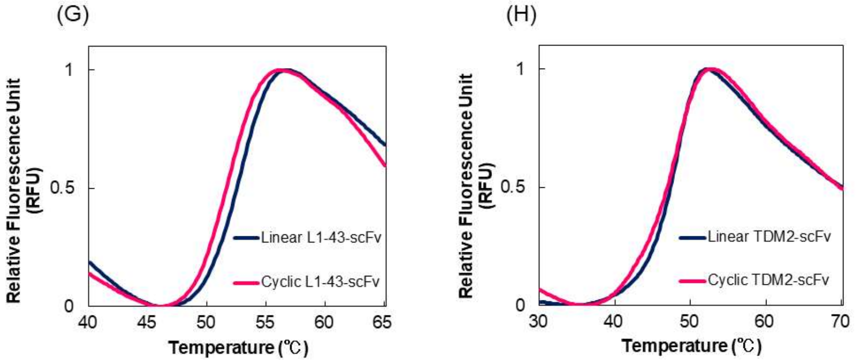

Figure S7. SDS-PAGE analyses of sortase A-mediated L1-43-scFv and TDM2-scFv cyclizations, and functional characterization of cyclic L1-43-scFv and cyclic TDM2-scFv.

(A) SDS-PAGE analysis of sortase A-mediated L1-43-scFv cyclization. Lane 1, sortase A; lane 2, L1-43-scFv-LPETG; lane 3, before sortase A reaction; lane 4, after the reaction at 25°C for 1 hour; lane 5, flow-through fraction of Ni-NTA affinity chromatography; lane 6, wash fraction; lane 7, elution fraction. (B) SDS-PAGE analysis of sortase A-mediated TDM2-scFv cyclization. Lane 1, sortase A; lane 2, TDM2-scFv-LPETG; lane 3, before sortase A reaction; lane 4, after the reaction at 25°C for 1 hour; lane 5, flow-through fraction of Ni-NTA affinity chromatography; lane 6, wash fraction; lane 7, elution fraction. (C) Molecular size distributions of L1-43-scFv by DLS analysis. Linear scFv (solid line) and cyclic scFv (dashed line) were concentrated to 3 mg/mL, and incubated at 4°C for 7 days. The DLS measurement was performed at 25°C. (D) Molecular size distributions of TDM2-scFv by DLS analysis. Linear scFv (solid line) and cyclic scFv (dashed line) were concentrated to 3 mg/mL, and incubated at 4°C for 7 days. The DLS measurement was performed at 25°C. (E) SPR sensorgrams of linear L1-43-scFv (left) and cyclic L1-43-scFv (right) at several scFv protein concentrations, and their K_D values. (F) SPR sensorgrams of linear TDM2-scFv (left) and cyclic TDM2-scFv (right) at several scFv protein concentrations, and their K_D values. (G) DSF analysis of L1-43-scFv at a heating rate of 1.0°C /min. (H) DSF analysis of TDM2-scFv at a heating rate of 1.0°C /min.

Figure S8

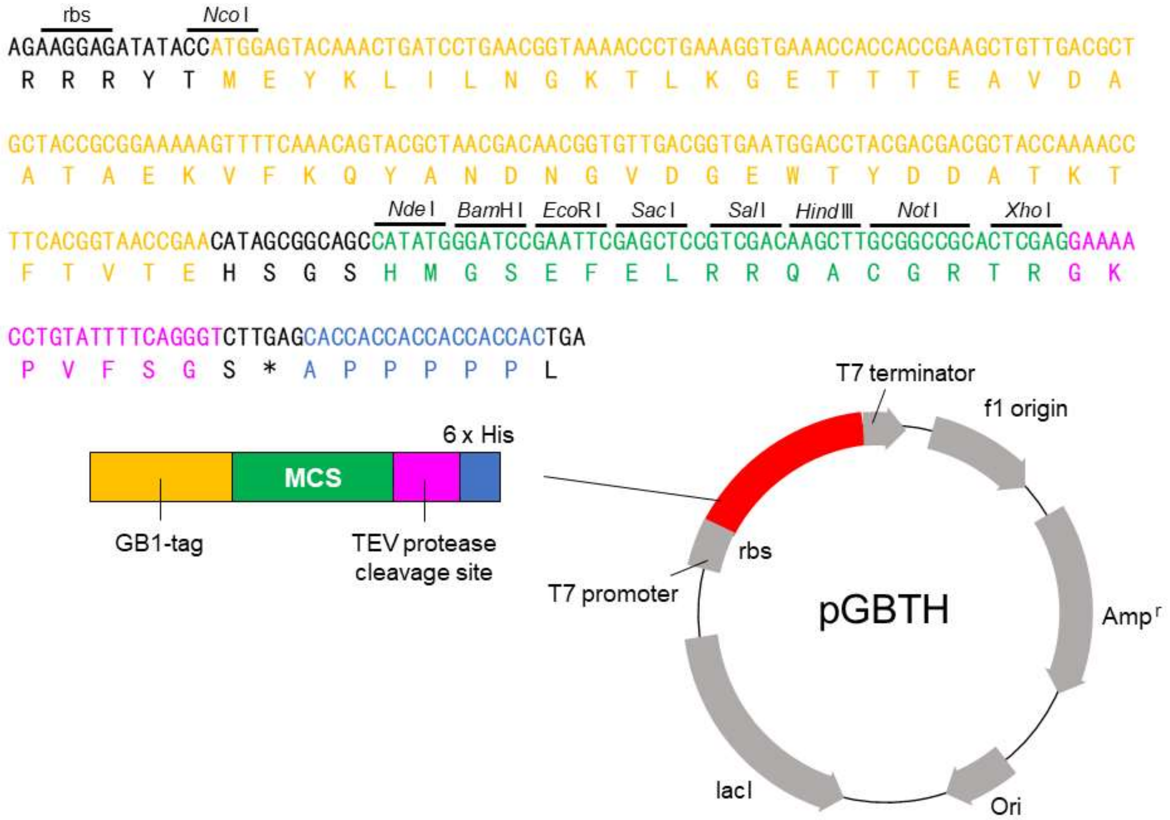

Figure S8. Plasmid map of pGBTH.
